# Pilot-Scale Airlift Bioreactor with Function-Enhanced Microbes for the Reduction of Refinery Excess Sludge

**DOI:** 10.3390/ijerph18136742

**Published:** 2021-06-23

**Authors:** Hongyan Mu, Min Zhang, Shanshan Sun, Zhaozheng Song, Yijing Luo, Zhongzhi Zhang, Qingzhe Jiang

**Affiliations:** 1 State Key Laboratory of Heavy Oil Processing, China University of Petroleum-Beijing, Beijing 102249, China; realzhangmin@126.com (M.Z.); sssun33@163.com (S.S.); song@cup.edu.cn (Z.S.); carole66@163.com (Y.L.); jiangqz@cup.edu.cn (Q.J.); 2 School of Petroleum Engineering, Yangtze University, Wuhan 430100, China

**Keywords:** refinery excess sludge, sludge reduction, function-enhanced microbes, airlift bioreactor

## Abstract

A pilot-scale airlift bioreactor (ALBR) system was built and operated continuously for refinery excess sludge (RES) reduction. Combined ALBR and function-enhanced microbes (composed of photosynthetic bacteria and yeast) were integrated into the system. The pilot-scale ALBR was operated for 62 days, and the start-up time was 7 d. Continuous operation showed that the sludge reduction efficiency was more than 56.22%, and the water quality of the effluent was satisfactory. This study focused on investigating the effects of hydraulic retention time (HRT) on the stability of the system and the effect of sludge reduction. Under different HRT conditions of 40, 26.7, 20, and 16 h, the sludge reduction rates reached 56.22%, 73.24%, 74.09%, and 69.64%, respectively. The removal rates of chemical oxygen demand (*COD*) and total nitrogen (TN) decreased with decreasing HRT, whereas the removal rate of NH_4_^+^-N increased. The removal rate of total phosphorus (TP) was approximately 30%. Results indicate that the ALBR and function-enhanced microbe system can reduce sludge and treat sewage simultaneously, and the effluent is up to the national emission standard. Addition of function-enhanced microbes can promote the degradation of petroleum hydrocarbon substances in the sludge, especially alkanes with low carbon numbers. This study suggests that the optimal HRT for the system is 16 h. The total operation cost of the ALBR combined with the function-enhanced microbe system can be reduced by 50% compared with the cost of direct treatment of the RES system.

## 1. Introduction

Refineries produce large amounts of sludge throughout the entire process of crude oil exploration until final refinement [1]. Oily sludge from petrochemical refineries is a state-specified hazardous waste that has been included in the National Catalogue of Hazardous Wastes in China (2016 edition). Refinery excess sludge (RES) accounts for 0.3–0.5% of the total treatment wastewater quantity, the moisture content is usually approximately 95%, and the production in China is 300 × 10^4^ t/y [2]. Moreover, the cost of sewage sludge treatment and disposal is over half of the total cost of wastewater treatment plants [3]. The sludge usually contains considerable amounts of toxic and harmful substances, such as parasite eggs, pathogenic microorganisms, heavy metals, and unstabilized organic matter [4,5]. If sewage sludge directly enters the ecological environment without proper treatment and disposal, it will cause secondary pollution and pose a serious threat to human health. Therefore, developing methods for sludge stabilization and mass reduction is of utmost importance, and how to reduce the sludge production during wastewater treatment is an increasing concern. Different types of sludge treatment techniques, including mechanical (e.g., ultrasonic disintegration, homogenization), thermal (e.g., high- and low-temperature hydrolysis), chemical (e.g., hydrolysis with oxygen, ozone, or sodium hydroxide), and biological (e.g., application of enzymes, cryptic growth) methods, have been developed [6,7]. Among these technologies, biological methods are considered the most effective approach from the economic and environmental standpoints.

Lysis-cryptic growth is an ideal method used to reduce excess sludge production during wastewater treatment. Cryptic growth refers to the growth of a secondary matrix formed by dead bacteria [8]. It consists of two steps, namely, cytolysis and the actual formation of the secondary matrix; of these, the first step is the limiting step. In the traditional model, promoting cell lysis increases the rate of cell decay, which can reduce the yield of residual sludge. Sludge lysis-cryptic growth could be enhanced using physical, chemical, and combined methods to reduce sludge production; some of these methods include ozone oxidation, chlorination, the thermophilic bacteria method, the high-temperature/high-pressure water method, thermal/ultrasonic treatment, and the function-enhanced microbe method [9]. *Pseudomonas* and *Aeromonas* species are psychrophilic bacteria that can increase the sludge reduction rates by four to eight times under aerobic conditions at 4–30 °C [10]. Sludge-lysing strains have been utilized in thermophilic aerobic digestion for waste-activated sludge, with a total suspended solid (TSS) removal rate of 32.8% [11]. Ultrasonic lysis-cryptic growth technologies use sequencing batch reactor activated sludge process (SBR) reactors to achieve sludge reduction rates of up to 50% [12]. Chlorination is usually employed to reduce excess sludge in the activated sludge process; this step can achieve sludge reduction rates of nearly 65%, but significant increases in SCOD (solluted chemical oxygen demand) have been observed [13]. Several methods considered to have potential sludge reduction method ability have been investigated and compared, and the use of function-enhanced microbes has been found to be the optimal solution from the viewpoint of cost performance [14]. However, if this method is to be expanded beyond the pilot scale and employed in industrial applications, the process design, operating procedures, and determination of function-enhanced microbes should be investigated further.

However, these methods are mainly applied to municipal excess sludge treatment and have not been applied to refinery excess sludge treatment. A previous work [15] showed that a functional mixture of yeast and photosynthetic bacteria (PSB) has a certain refinery-excess-sludge-reducing effect. PSB have been used since the 1960s to treat heavy metals and all types of industrial sewage. PSB have also been successfully adopted to treat various wastewater types and increase the removal efficiency of *COD* (chemical oxygen demand), N, and P [16,17,18]. Membrane bioreactors (MBRs) and sludge disintegration systems can be used to prevent excess sludge production during wastewater treatment, and the disintegration sludge may be recycled to the MBR as a feed solution [19]. Nevertheless, the feasibility of a combined membrane bioreactor and function-enhanced microbe method for RES reduction based on lysis-cryptic growth still needs further investigation.

In this study, a pilot-scale airlift bioreactor (ALBR) system with a capacity of 6 m^3^/d was built in a refinery wastewater treatment plant. Combined ALBR and function-enhanced microbes were integrated into the system used for RES reduction. The system was carried out in a continuous operation. The efficiencies of a RES and sewage treatment system were investigated. The key objectives of this work are: (a) to evaluate the ALBR system start-up time and operating stability; (b) to determine changes in RES reduction rate as a function of the hydraulic retention time (HRT); (c) to assess *COD*, NH_4_^+^-N, total nitrogen (TN), and total phosphorus (TP) contents; and (d) to determine petroleum hydrocarbon substances in the resulting sludge. The result provides a good reference for RES reduction system process optimization in the future. The results here will benefit further studies and actual applications.

## 2. Materials and Methods

### 2.1. Refinery Excess Sludge

The RES sample was obtained from the secondary sedimentation tank of a refinery sewage treatment plant. This plant treats refinery sewage via the activated sludge method. The *COD*, NH_4_^+^-N, TN, TP, suspended solid (*SS*), and pH levels observed after sedimentation were 30–85 mg/L, 5.0 mg/L, 6.0 mg/L, 1.0 mg/L, 20.0–200.0 mg/L, and 8.0–9.0, respectively.

### 2.2. Microbes

The function-enhanced microbes were composed of PSB and yeast. The PSB were screened and cultured from the sludge of a photo-anaerobic reactor in a research laboratory and found to be *Rhodopseudomonas* sp. The culture conditions of the PSB included a temperature of 26–30 °C, a light intensity of 1000–3000 lx, and pH 7–7.2. The yeast used in this study was mainly composed of *Candida* sp. and *Rhodotorula* sp., which were from the mixed bacteria preserved in our laboratory.

### 2.3. ALBR System Pilot Plant Description

#### 2.3.1. ALBR Configuration

**Figure 1:**
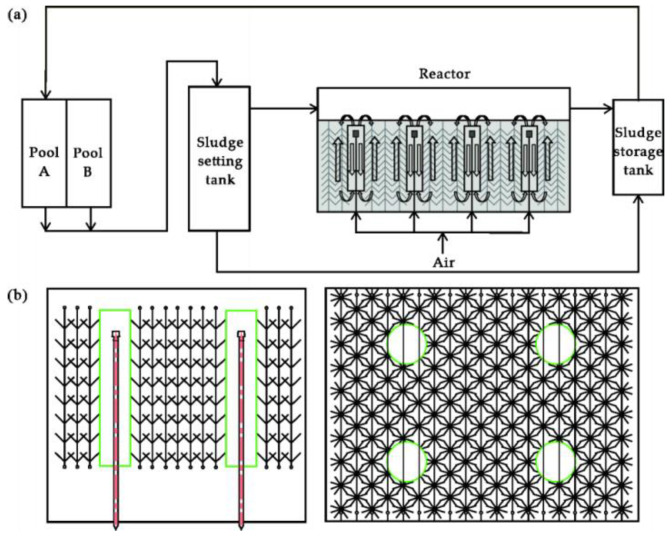
Schematic diagram of the ALBR and function-enhanced microbe system. (**a**) Schematic diagram. (**b**) Elastic solid filler.

Figure 1 shows a schematic diagram of the ALBR system. The ALBR features (Figure 1a) an internal loop aerobic biofilm reactor. The volume of the ALBR is 4 m^3^, and its size is 2.2 m × 1.2 m × 1.2 m. Air for the aeration of the ALBR is taken from the purified compressed air in the plant, and the air pressure is 0.2 MPa. The inlet flow is regulated by a gas-pressure-reducing valve and glass air rotameter. Valves are installed on each branch of the inlet pipeline to ensure uniform flow in the aeration heads. The elastic solid filler (Figure 1b) is made of polymer and added with an antioxidant, hydrophilic agent, stabilizer, adsorbent, and other additives. The length of the filler is 1400 mm, and the radius of the fibers is 80 mm.

#### 2.3.2. ALBR Operation

At the beginning of the experiment, 1 m^3^ of activated sludge from the outlet of a section of the A^2^O (anaerobic-anoxic-oxic) unit in the biochemical section of the sewage treatment plant was injected into the ALBR with MLSS (mixed liquor suspended solids) of 2532 mg/L. Then, 3 m^3^ of effluent from pool A (Figure 1) was injected into the ALBR. Nutrient solution was prepared from glucose and urea potassium dihydrogen phosphate with a *COD*/N/P ratio of 100:5:1. Aeration was initiated, and the ratio of gas to water was controlled to 15:1. The sewage, sludge, and nutrient solution were mixed evenly. The inoculation amount of function-enhanced microbes was 1/4 of the effective pool volume. The ALBR temperature was 20–30 °C (environment temperature), and the pH was maintained at 7.0 ± 0.2 over the entire test period. HCl was used to adjust the influent pH when necessary. The HRT was adjusted by controlling the inflow velocity. The HRTs were 16, 20, 26.7, and 40 h for influent flows of 250, 200, 150, and 100 L/h, respectively.

### 2.4. Analytical Methods

The pH and the dissolved oxygen (DO) content of the ALBR were monitored regularly using a pH meter and a DO meter, respectively. *COD*, NH_4_^+^-N, TP, TN, and *SS* were measured using standard methods [20]. The operation of some water-quality characterization items can refer to the literature [21,22]. The temperature was recorded as the water temperature in the bioreactors as measured by a thermometer. In order to analyze the collected sample, a GC-MS (gas chromatography-mass spectrometer) (Thermo Finnigan Trace DSQ, Waltham, MA, USA) was utilized.

Sludge yield calculation: sludge production is related to the inlet *SS*, outlet *SS*, sludge discharge at the bottom of the reactor, sludge discharge at the bottom of the secondary sink pool, and *COD* removal rate.

(1) $$M=\frac{\left(SS_{1}-SS_{2}\right)\times V-M_{1}-M_{2}}{\left(COD_{1}-COD_{2}\right)\times V}$$

where *M* is the sludge production per 1 kg of *COD*, *COD*_1_ is the *COD* in the inlet water, *COD*_2_ is the *COD* in the outlet water, *SS*_1_ is the suspended matter concentration in the inlet water, *SS*_2_ is the suspended matter concentration in the outlet water, *V* is the treated water volume, *M*_1_ is the sludge discharge at the bottom of the reactor, and *M*_2_ is the sludge discharge at the bottom of the secondary sedimentation tank. 

## 3. Results and Discussion

### 3.1. ALBR Performance

The ALBR system was operated for 62 d, the first 7 of which represent the start-up phase. The stability of the system under various HRT conditions and the optimal conditions for *COD* removal were then investigated.

#### 3.1.1. ALBR System Start-Up

**Figure 2:**
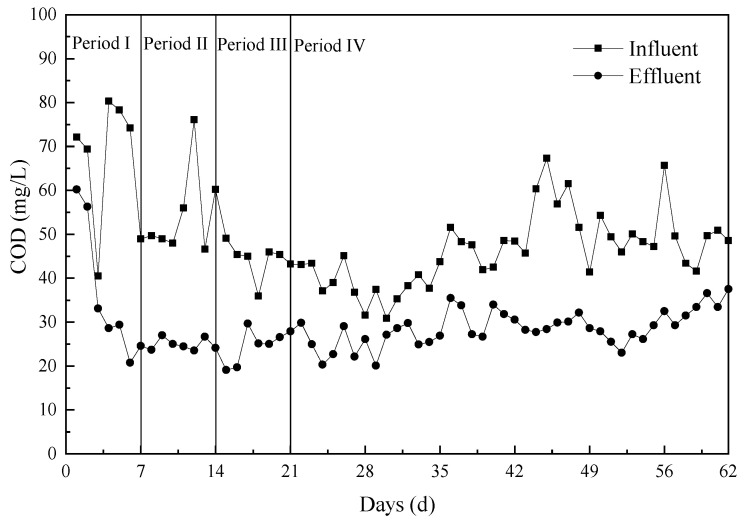
ALBR performance data for different periods.

The ALBR system was quickly initiated and operated for 7 d (Figure 2). After operation for 7 d, *COD* concentrations in the effluent mostly remained below 30 mg/L, thus meeting the national drainage standard. The start-up period of the proposed system was shorter than those reported [5,16,23]. The significant difference in start-up times obtained between this work and other studies may be attributed to the inoculation of high concentrations of the mixed culture at the beginning of the start-up period in the present work.

#### 3.1.2. Operating Stability of the ALBR System 

The stability of a system is very important in industrial applications. HRTs remarkably affect a pollutant’s removal efficiency. The RES reduction performance of the ALBR under different HRTs was assessed. During the pilot-scale treatment, the performance of the system was investigated under different influent *COD* concentrations and environmental factors.

The different HRTs were treated as dissimilar periods as follows (Figure 2).

Period I (1–7 d): (HRT = 40 h) During start-up, the inoculation amount was 25% of the effective pool capacity of the ALBR. At the sludge culture stage, the *COD*/N/P ratio remained relatively stable at 100:5:1 via the addition of nutrient solution (20 L/d) prepared from glucose and potassium dihydrogen phosphate in urea. The DO was controlled to 3 mg/L. After 2 d of culture, a slick yellowish biofilm formed on the surface of the filler, and the sludge culture phase ended. Because of its good applicability, the process can be started quickly without acclimation. 

HRT is regulated by the wastewater influent. The initial wastewater influent was 100 L/h, the HRT was 40 h, and the organic volume load was 48 g *COD*/m^3^·d. After operation for 7 d with an HRT of 40 h, the average influent *COD* was 66.3 ± 15.34 mg/L and the effluent COD was below 30 mg/L. The thickness of the yellowish biofilm on the filler gradually increased, and the color changed from pale yellow to reddish brown. PSB tend to form biofilms on abiotic surfaces under a wide range of environmental conditions [24]. The ALBR system was successfully started. 

Period II (8–14 d): (HRT = 26.7 h) The ALBR system temperature varied with the ambient temperature, which was approximately 20–30 °C. The DO was controlled to 5–6 mg/L. The initial wastewater influent was 150 L/h, and the HRT was 26.7 h. After 7 d of operation with an HRT of 26.7 h, the average influent *COD* was 55.1 ± 10.45 mg/L and the average effluent *COD* was 24.9 mg/L. The effluent obtained was basically identical to that recovered from the A^2^O process of a sewage treatment plant. Concentrated HCl was added to the influent to adjust the pH of the wastewater to 7 as determined for continuous reactor operation.

Period III (15–21 d): (HRT = 20 h) The initial wastewater influent was 200 L/h, and the HRT was 20 h. After 7 d of operation with an HRT of 20 h, the average influent *COD* was 44.3 ± 4.1 mg/L and the average effluent *COD* was 24.7 ± 3.97 mg/L. 

Period IV (22–62 d): (HRT = 16 h) The initial wastewater influent was 250 L/h, and the HRT was 16 h. In periods I–IV, various suspension parameters, such as DO, pH, and other environmental conditions, remained nearly constant over the entire experimental period. After 7 d of operation with an HRT of 16 h, the average influent *COD* was 39.4 ± 4.75 and the average effluent *COD* was 25 ± 3.56 mg/L. The HRT set at this stage is identical to the HRT designed by the original A^2^O process. The effluent water quality remained below 30 mg/L, which meets the corresponding standard. The stability and sludge reduction effect of the system operated under an HRT of 16 h was continuously measured for 34 d, and the average influent and effluent *COD*s were found to be 47.7 ± 8.17 mg/L and 29.4 ± 3.82 mg/L, respectively. The equipment was run continuously for 62 d and showed stable operation.

### 3.2. Refinery Excess Sludge Reduction

**Figure 3:**
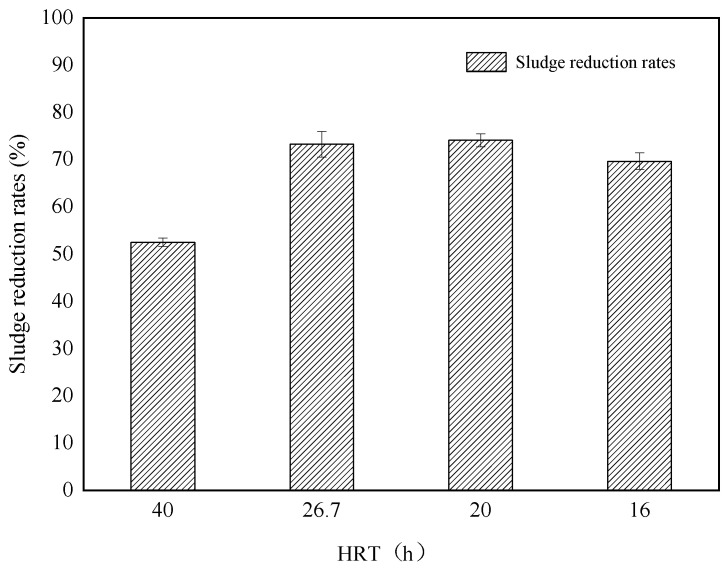
Sludge reduction rates at different HRTs.

Sludge reduction rates are an index of the average sludge reduction effect. The reduction rates of the ALBR are related to the amount of sludge in and out of the system, the biomass produced by *COD* removal, the amount of sludge discharged from the secondary sedimentation tank, and the amount of sludge discharged from the bottom of the reactor. Addition of function-enhanced microbes to the ALBR could reduce sludge by over 50% under different HRT conditions (Figure 3). The sludge reduction rates increased with increasing HRT. When HRTs of 16, 20, 26.7, and 40 h were applied, sludge reduction rates of 69.64%, 74.09%, 73.24%, and 52.49% were obtained, respectively. As the HRT decreased, water inflow increased, the contents of suspended solids and dissolved organic matter increased, and the sludge reduction rates decreased. When the HRT was 40 h, the sludge reduction rates were 52.49%, likely because of the start-up reaction. At this stage, sludge depositions at the bottom of the reactor and secondary sedimentation tank were observed, leading to low calculation results. The laboratory-scale results [15] of the addition of functional microbes showed that the MLSS removal of sludge could reach 56.2% after 7 d of continuous operation, and the MLSS was measured to evaluate the effect of sludge reduction. Comparison of the present findings with laboratory results could confirm that the feasibility of using the ALBR with added functional microbes could be confirmed.

Reductions in sludge may be explained as follows. First, function-enhanced microbes promote sludge degradation by enhancing the breakage of cell walls, which is the rate-controlling step of recessive growth. Because the cell wall is composed of cellulose, lignin, and other refractory components, yeast can secrete extracellular enzymes to break dead cells in the sludge; dissolve protein, fat, polysaccharides, and other intracellular substances; and then hydrolyze and acidify them to further decompose them into fatty acids and lipids and other biodegradable organic matter. PSB can also use organic matter for self-reproduction [25] and convert organic matter into energy and gas under aerobic conditions to achieve sludge reduction. Second, the internal circulation function of the MBR can improve the utilization rate of oxygen, increase the efficiency of gas-liquid mass transfer [26], quickly establish the dominant flora of the system, accelerate the reproduction of function-enhanced microbes, and use dissolved cell materials as a substrate for regrowth. Third, biological feeding occurs in the later stages of the reaction. Protozoans, which were detected in the laboratory test [15], could extend the food chain by biological predation, so that sludge converted into energy and in the food chain transmission process loss could achieve sludge reduction.

**Figure 4:**
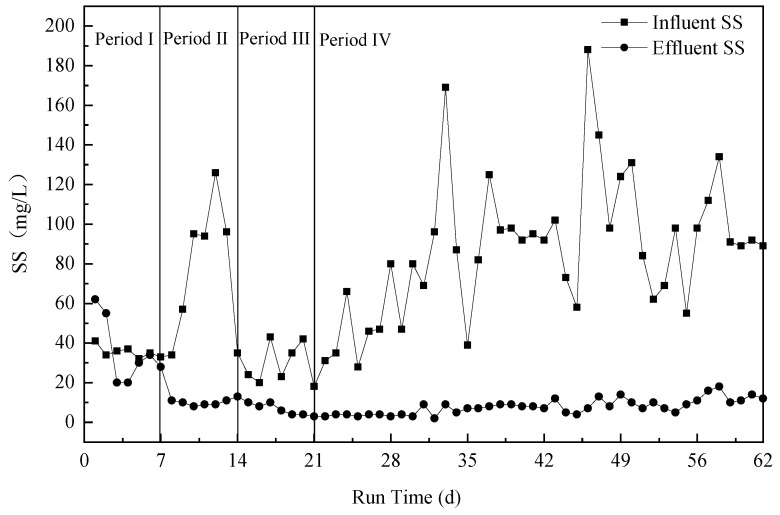
*SS* change under different run times in the ALBR system.

Because of the remarkable change in sludge settling performance in the A^2^O process in the sewage treatment plant, the *SS* concentration in the ALBR influent revealed notable changes (Figure 4). The ALBR may be likened to a biofilter reactor that intercepts the sludge carried by the influent and digests it to obtain the necessary nutrients. Therefore, the SS of the effluent was relatively stable and remained below 10 mg/L.

The results of this study thus far indicate that the addition of functional bacteria can effectively degrade the organic matter in the sludge and achieve the reduction goal effectively. When the ALBR reaction system is under stable operation, the optimum HRT is 16 h, the reduction rate could reach 69.64%, and the effluent quality is stable. The reduction process is based on the recessive growth of lysosomes of functional microbes, which is caused by microbial strengthening and extension of the sludge age.

### 3.3. COD, NH_4_^+^-N, TN, and TP Removal

**Figure 5:**
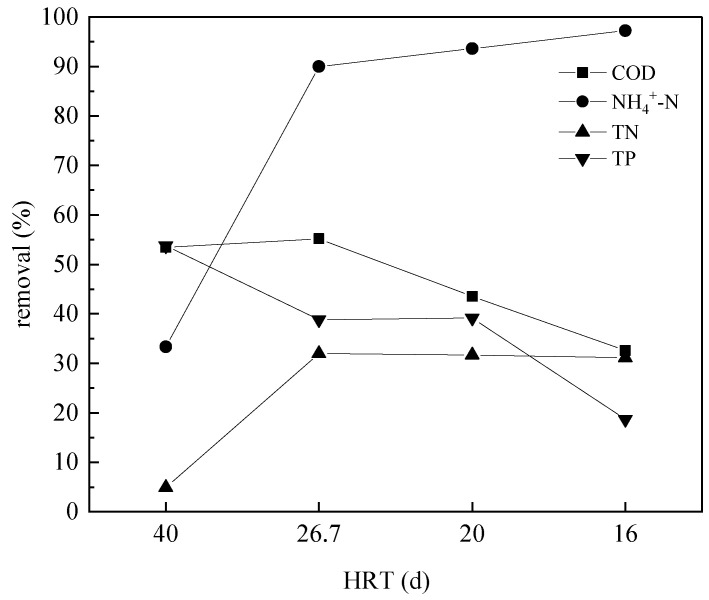
*COD*, NH_4_^+^-N, TN, and TP removal under different HRTs in ALBR system RES reduction.

COD is an index of total organic compounds. TN is a representative index of organic and inorganic nitrogen, and phosphorus is an important pollutant in wastewater. NH_4_^+^-N exists in water in the form of free ammonia or ammonium salt and is mainly derived from the decomposition products of nitrogen-containing organic compounds in sewage under the action of microbes. Thus, *COD*, NH_4_^+^-N, TN, and TP removal rates were determined during sludge reduction (Figure 5).

At different HRTs, the ALBR reduced the contents of organic matter remarkably, yielding *COD* removal rates of 53.4–32.6%. The removal rate of *COD* decreased with decreasing HRT, likely because of the mobilization of organic matter in the wastewater. *COD* removal from wastewater is largely related to VFA (volatile fatty acid) removal [27]. With the decrease of HRT, the treatment time of wastewater is shortened, which leads to the increase of *COD* in wastewater. Although the biomass concentration in the ALBR is related to different factors (e.g., light intensity, HRT, influent characteristics) [28], the removal of *COD* is comparable to that observed in previous research [29]. 

During sludge reduction, the dissolution of sludge cells occurs, and this process releases proteins, nucleic acids, and polysaccharides, thereby increasing the effluent ammonia nitrogen, TN, and TP. Thus, the removal rates of ammonia nitrogen, TN, and TP are also within the scope of the present investigation. The ALBR could clearly remove ammonia nitrogen from the sludge. The removal rates of ammonia nitrogen when HRTs of 16, 20, 26.7 and 40 h were applied were 97.2%, 93.58%, 89.98%, and 33.33%, respectively. The influent ammonia nitrogen concentration was approximately 5 mg/L. When the HRT was 40 h, the removal efficiency of ammonia nitrogen was fairly low on account of the start-up characteristics of the reactor. When the reactor was run for 1 week, the removal efficiency of ammonia nitrogen increased to ~90%. As the reactor was operated further, the removal rate of ammonia nitrogen gradually increased. When the HRT was 16 h, the removal rate of ammonia nitrogen reached 97.2%. However, the effluent ammonia nitrogen concentration was only approximately 0.2 mg/L, which is in line with the national emission standard. The influent TN concentration was 6 mg/L, and the TN removal rate decreased by 53.7%, 38.8%, 39.2%, and 18.7% as the HRT decreased. Decreases in HRT increase the influent and the total *SS* and *COD* concentrations, thereby resulting in an increase in organic load and changes in TN removal efficiency. The influent TP concentration was approximately 1 mg/L. The reactor could remove TP but only at a low rate of ~30%, which indicates that the reduction of sludge by the reactor does not cause increase in the TP. During sludge reduction, the concentrations of ammonia nitrogen, TN, and TP in the effluent were lower than those in the influent, and the *COD* in the effluent remained stable. These data indicate that the organic matter released by sludge reduction is completely utilized without any impact on the reactor. Given the efficiency of *COD* removal and ammonia oxidization, the proposed treatment may be concluded to have no negative impact on the performance of the ALBR. The reactor can reduce sludge and treat sewage simultaneously.

### 3.4. Degradation of Crude Oil Components

**Figure 6:**
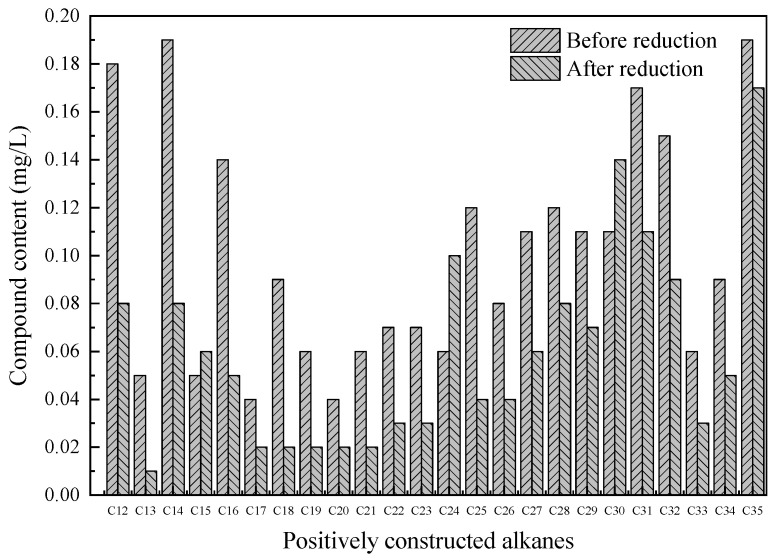
Compound content of positively constructed alkanes before and after reduction.

**Figure 7:**
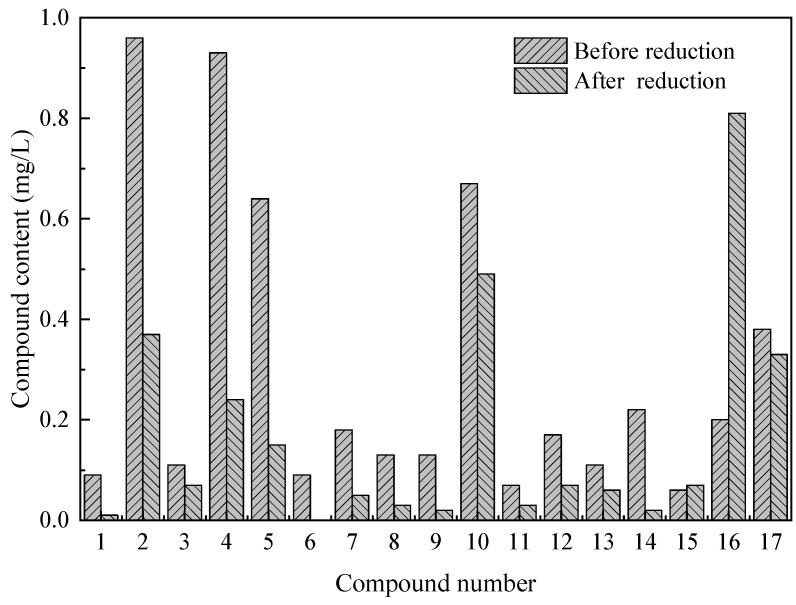
Contents of certain compounds (listed below) before and after reduction (1—C_8_H_10_; 2—C_6_H_4_C_l2_; 3—C_8_H_8_O; 4—C_2_Cl_6_; 5—C_8_H_10_O; 6—C_10_H_8_; 7—C_9_H_12_O; 8—C_7_H_5_NS; 9—C_9_H_12_O; 10—C_10_H_14_; 11—C_14_H_20_O_2_; 12—C_14_H_26_O_2_; 13—C_16_H_22_O_4_; 14—C_15_H_25_N; 15—C_16_H_23_N; 16—C_18_H_35_NO; 17—C_23_H_32_O_2_).

The oil components before and after reduction were analyzed (Figure 6 and Figure 7). GC-MS demonstrated that the main organic matter in excess sludge are n-alkanes. Because the oily components in the excess sludge mainly come from petroleum, the product of the GC-MS peak area percentage and oil content could be used to express the contents of linear alkanes. Significant changes in the distribution characteristics of petroleum hydrocarbons during excess sludge reduction were observed (Figure 6). In particular, the contents of n-alkanes decreased significantly. From carbon 12 to carbon 35, the lower is the carbon number, the easier it is to degrade. Organic compounds with low molecular weights and simple structures can easily be degraded. The yeast used in this study was mainly composed of *Candida* and *Rhodotorula*, which can oxidize C_9_–C_25_ n-alkanes and be used as a carbon source for growth. GC-MS analysis found that it also contains phenols, alcohols, ketones, esters, and other compounds, mainly benzene ring compounds, and it changes before and after reduction (Figure 7). The contents of linear alkanes remarkably decreased, but other compounds were hardly degraded (Figure 7). Alkanes, which feature simple molecular structures, are relatively easy to degrade. After the introduction of functional microbes, the sludge could be reduced, and the petroleum hydrocarbons in the sludge could be degraded simultaneously.

### 3.5. Optimization of System Process for Engineering Applications

**Figure 8:**
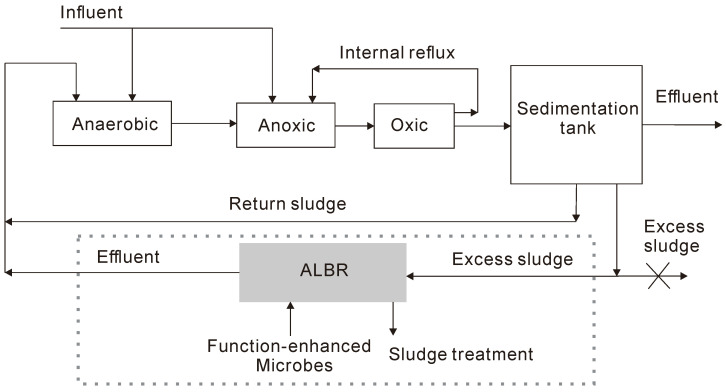
Treatment flow chart of refinery wastewater process optimization.

The feasibility of the ALBR combined with functional microorganisms for the reduction of RES was verified by the pilot test. Thus, the proposed refinery wastewater treatment process was optimized (Figure 8). In the existing sewage treatment process, the excess sludge produced in the settling tank cannot be discharged; in this case, the ALBR combined with functional microbes could be added to reduce the sludge (Figure 8, dotted portion). The reduced sludge can then be subjected to further treatment, such as sludge thickening. The cost of mud cake treatment is approximately 670 yuan/ton, and the cost per ton can be reduced by ~50% when the proposed technology is applied. Considering that the function-enhanced microbes added to the ALBR are composed of PSB and yeast, the excess sludge can be recycled. Coenzyme Q10, carotenoids, bacteriochlorophylls, 5-aminolevulinic acid, and proteins [30] can be extracted from PSB cells, and the remnant sludge can be reused as fertilizer.

## 4. Conclusions

A pilot-scale ALBR system with a capacity of 6 m^3^/d was built and operated continuously for RES reduction. Combined ALBR and function-enhanced microbes were integrated into the system. Continuous operation showed that the sludge reduction efficiency was higher than 56.22%, and the water quality of the effluent was satisfactory. The outcomes of this research include the following findings:

1. The pilot-scale ALBR was operated for 62 days, and the start-up time was 7 d.
2. Under different HRT conditions of 40, 26.7, 20 and 16 h, the sludge reduction rates reached 56.22%, 73.24%, 74.09%, and 69.64%, respectively.
3. The removal rate of *COD* and TN decreased with decreasing HRT, while the removal rate of NH_4_^+^-N increased. The removal rate of TP was approximately 30%.

In summary, the results indicate that the ALBR and function-enhanced microbe system can reduce sludge and treat sewage simultaneously, and the effluent is up to the national emission standard. The addition of function-enhanced microbes can promote the degradation of petroleum hydrocarbon substances in the sludge, especially alkanes with low carbon numbers. This study suggests that the optimal HRT for the system is 16 h. The total operation cost of the ALBR combined with the function-enhanced microbe system can be reduced by 50% compared with the cost of direct treatment of RES.

## Data Availability

Data is contained within the article, the data presented in this study are available in this article.
